# Neurocircuit differences between memory traces of persistent hypoactivity and freezing following fear conditioning among the amygdala, hippocampus, and prefrontal cortex

**DOI:** 10.3934/Neuroscience.2021010

**Published:** 2021-01-19

**Authors:** Masatoshi Takita, Yumi Izawa-Sugaya

**Affiliations:** 1 Human Informatics and Interaction Research Institute, National Institute of Advanced Industrial Science and Technology (AIST), Ibaraki 305-8566, Japan; 2 Center for Neuroscience and Biomedical Engineering, The University of Electro-Communications, Tokyo, Japan; 3 Graduate School of Life and Environmental Sciences, University of Tsukuba, Ibaraki, Japan

**Keywords:** extinction, footshock intensity, freezing, ibotenic acid, motor activity, rats

## Abstract

We aimed to investigate the persistent trace of one traumatic event on neurocircuit controls in rats. Conditioning was reflected by reductions in rates of ‘freezing’ and ‘other-than-freezing’ motor activities, between which rats could alternate on delivery of pulsed footshocks of intensity 0.5 mA but not 1.0 mA. At the latter intensity, freezing began to suppress motor activity. The conditional responses evident during both the context and tone sessions persisted when the tests were repeated on post-conditioning days 7 and 8. Thus, difficulties with fear extinction/reduction remained. However, persistence was not evident on post-conditioning days 1 and 2. One day after the 1.0 mA pulsed footshock, ibotenate lesions and corresponding sham surgeries were performed in unilateral and bilateral hemispheres of the amygdala, hippocampus, and prefrontal cortex, as well as three different disconnections (one unilateral and another contralateral lesions out of three regions, a total of nine groups), and were tested on days 7–8. The drastic restoration of freezing following bilateral amygdala lesions was also evident in animals with three types of disconnection; however, this was not the case for hypoactivity. These results imply that a serious experience can drive different neurocircuits that all involve the amygdala, forming persistent concurrent memories of explicit (e.g., ‘freezing’) or implicit (e.g., ‘other-than-freezing’ motor activity) emotions, which may exhibit mutual interference.

## 1. Introduction

Post-traumatic stress disorder (PTSD) has been studied in comparison with conditioned fear and reduction thereof in humans and animals [1]. Memory processes for acquisition and reduction of fear reportedly obey the general processes of learning and memory [2],[3]; the reduction shares similarities with putative learning habituation [4]. One definition of habituation is the following: “The weaker the stimulus, the more rapid and/or more pronounced is habituation. Strong stimuli may yield no significant habituation” [5]. Indeed, weaker unconditioned stimuli were ineffective in terms of inducing freezing, being rather associated with the movement [6]. This implies that the persistent mechanism for conditioned fear must be studied independently to understand the clinical symptoms of PTSD.

In daily life, emotions that have been categorized separately can coexist with one another (cf. [7]). The coexistence of negative emotions has been suggested in patients with PTSD [8], which involves the amygdala and other limbic structures [9]. Indeed, several different types of medicine are prescribed for regulation of these emotional states in PTSD [10], implying that the amygdala is a cross point for trafficking complex emotions related to the symptoms of PTSD. Here, we targeted both freezing and other-than-freezing activity; these behaviors alternate [11].

Fear conditioning in rats involves the amygdala via the thalamus or the modality-relevant cortex [12]. The acquisition and retention processes of conditioning also involve the hippocampus [13]–[15] and the medial prefrontal cortex (mPFC) [16]–[19]. These three brain regions, which are directly interconnected, have also been implicated in PTSD [9]. The basomedial/basolateral complex of the amygdala projects reciprocally to the ipsilateral caudal/ventral hippocampus [20]–[25] and to the deep layers of the mPFC [20],[26]–[30]; the posterior region of the complex terminates in the mPFC, which terminates in the anterior region of the complex [31],[32]. The hippocampus unidirectionally projects to the ipsilateral mPFC [33],[34]; for review, [35]. Direct descending projections from the mPFC to the hippocampus have not been reported. These anatomical relationships also likely control not only fear [36] but also anxiety [37].

By referring to previous model studies of impaired fear reduction [38], we investigated the effects of a single serious event on changes in the neurocircuitry of the amygdala, hippocampus, and prefrontal cortex. We initially examined the roles played by each brain region following the creation of bilateral lesions using ibotenic acid. We then explored functional connectivity among the three regions using the disconnection method (one unilateral and another contralateral lesions out of three regions). We also created unilateral lesions in each brain region.

We hypothesized that: (1) all lesions would similarly affect activity and freezing and (2) the behavioral indices would be mutually complementary; amygdala-centered networks would involve the hippocampus and prefrontal cortex.

## 2. Materials and methods

### 2.1. Animals

Seven-week-old male Sprague Dawley rats were purchased from Charles River (Tokyo, Japan; n = 164) and housed in a temperature-controlled room (24 ± 1°C) with a 12 h light/dark cycle. All experimental procedures and the care of laboratory animals were approved by the Animal Ethical Committee of the National Institute of Advanced Industrial Science and Technology in accordance with the National Institutes of Health Guide for the Care and Use of Laboratory Animals (NIH Publication No. 80–23, revised in 1996).

### 2.2. Apparatus

A double-plywood soundproof container (internal dimensions, 55 × 70 × 60 cm) was equipped with fan aeration and a 10 W light. Two speakers provided constant white noise and an occasional pure tone at 75 dB (1 kHz). An acrylic chamber (25 × 31 × 30 cm; Neuroscience Inc., Tokyo, Japan) was placed in the soundproof container to provide scrambled electric AC footshock pulses (50 Hz, 0.5 or 1.0 mA) through the 3 mm stainless steel rod flooring (16 mm center to center). Meaningless clip art covered one of the transparent chamber walls to visually enrich the environment. Rat activity was measured through motion on a soft rubber mat; an eddy-current sensor (±10 V range at 1 Hz; Keyence Inc., Osaka, Japan) recorded the motion of a metal spring attached to the outside of the chamber. Freezing was monitored through a CCD video camera. Desktop computers controlled the conditioning apparatus and were used for data storage. Between conditioning or test sessions, the chamber was cleaned with a 70% alcohol solution and dried.

### 2.3. Behavioral procedures

One week after purchase, each animal (240–305 g) was placed in the conditioning chamber and allowed to acclimate for 3 min. The conditioning stimulus, a 1 min pure tone, was then presented, and 12 footshock pulses were given with a 2 s on/off cycle with a 14 s delay. The ends of the tone and shock were simultaneous. Animals were returned to their cages 1 min after the tone/shock session. Each animal was tested in the chamber once a day on two consecutive test-retest days (D), D1–D2 or D7–D8, after conditioning with respect to context (first 3 min), tone (next 1 min without shock), and post-tone (over the next 6 min, two 3 min periods). Recorded activity was calculated from a 10 s moving average of the absolute amplitude of the eddy-current, normalized to body weight (mV/kg/min). Freezing was defined as the absence of any movement, and behavior was scored every 1 s as moving or not (contextual freezing for 3 min and tone-cued freezing for 7 min). To evaluate possible freezing reduction and to compare temporal properties between motor activity and freezing that frequently ran over tone duration, paw motion was indexed for freezing for the 6-min post-tone period.

### 2.4. Surgery and histology

Animals were tested on D7 and D8. Brain surgeries for the lesion study were performed one day after conditioning, as previously described [39]. Animals were anesthetized with pentobarbital (10–15 mg/kg, intraperitoneal) and sevoflurane (1.5–2%) and placed in a stereotaxic frame. Using a cannula (0.3 mm outer diameter) equipped with a 25 µL syringe driven by an infusion pump (CMA102; Carnegie Medicine, Sweden), ibotenic acid (10 µg/µL in 0.01 M phosphate buffer, pH 7.4; Tocris, Bristol, UK) or vehicle was infused at a rate of 0.1 µL/min with a 2 min post-infusion time into the following unilateral, bilateral, or contralateral target areas in a counterbalanced fashion: basolateral amygdala (0.15 µL; coordinates: 3.2 mm posterior and 4.9 mm lateral to the bregma, 7.3 mm below the dura), caudal-medial/ventral hippocampus (0.4 µL; coordinates: 5.8 mm posterior and 5.5 mm lateral to the bregma, 5.5 mm below the dura), or mPFC (0.1 µL; coordinates: 3.3 mm anterior and 0.8 mm lateral to the bregma, 3.6 mm below the dura) [40]. By combining a unilateral lesion in one brain area with a contralateral lesion in another brain area, the direct anatomical projection between the areas was disconnected in each brain hemisphere [41]. At the end of the experiment, brains were sectioned (70 µm) on a cold microtome and stained with thionin to verify lesion locations.

### 2.5. Data analysis and statistics

Analyses of variance (ANOVA) with post-hoc analyses and Mann-Whitney U-tests were performed offline using commercial software (StatView-J 5.0; HULINKS, Tokyo, Japan). The statistical criterion of significance was P < 0.05 for all analyses. All values are expressed as the mean ± standard error of the mean (SEM).

## 3. Results

### 3.1. Effect of fear conditioning shock intensity and test interval on motor activity and freezing

Three experimental groups (n = 5–6 rats per group) were separated on conditioning day (D0) into the following groups: tone with no shock (control group), tone with 0.5 mA pulsed footshock (0.5 mA group), and tone with 1.0 mA pulsed footshock (1.0 mA group). Each group was further separated for test-retest on D1–D2 or D7–D8.

**Figure 1.**
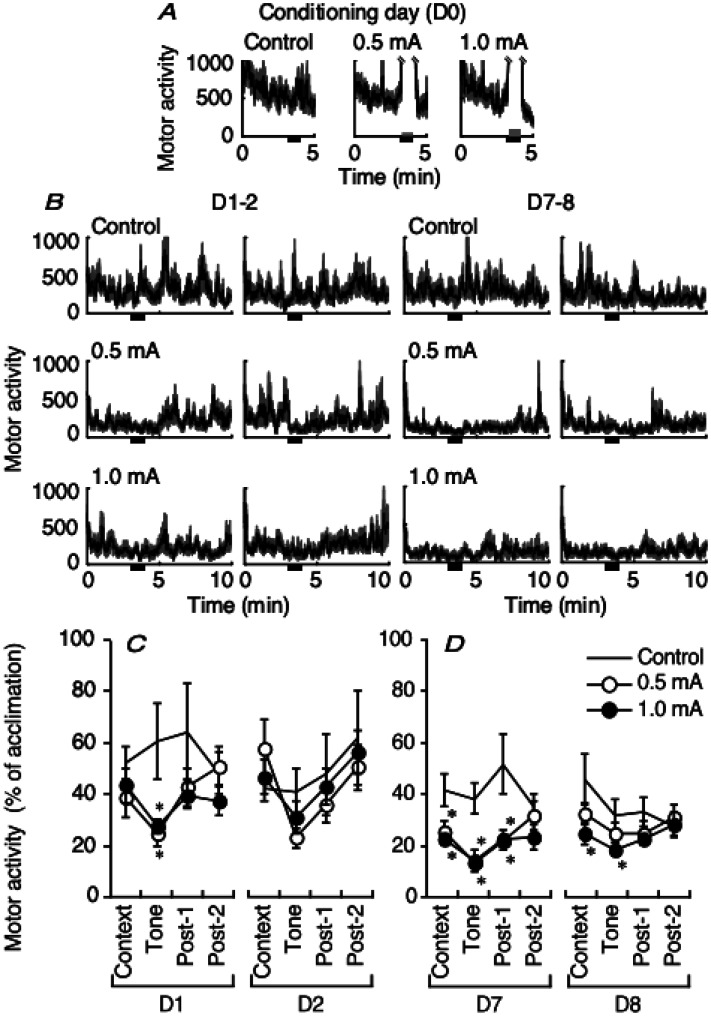
Time course of motor activity (mV/kg/min; mean and SEM in black and gray, respectively) during the tone period (black bar) with pulsed footshocks of 0.0, 0.5 (thin gray bar), or 1.0 Ma (thick gray bar) on the conditioning day (A) or test-retest days (B). During context, tone, and the two post-tone periods (Post-1 and Post-2; 3 min each) on D1–D2 (C) and D7–D8 (D), changes in motor activity (mean ± SEM) were normalized to the initial 3 min acclimation on D0 (n = 5–6; * indicates P < 0.05, post-hoc analysis following four-way ANOVE with repeated measures).

**Figure 2.**
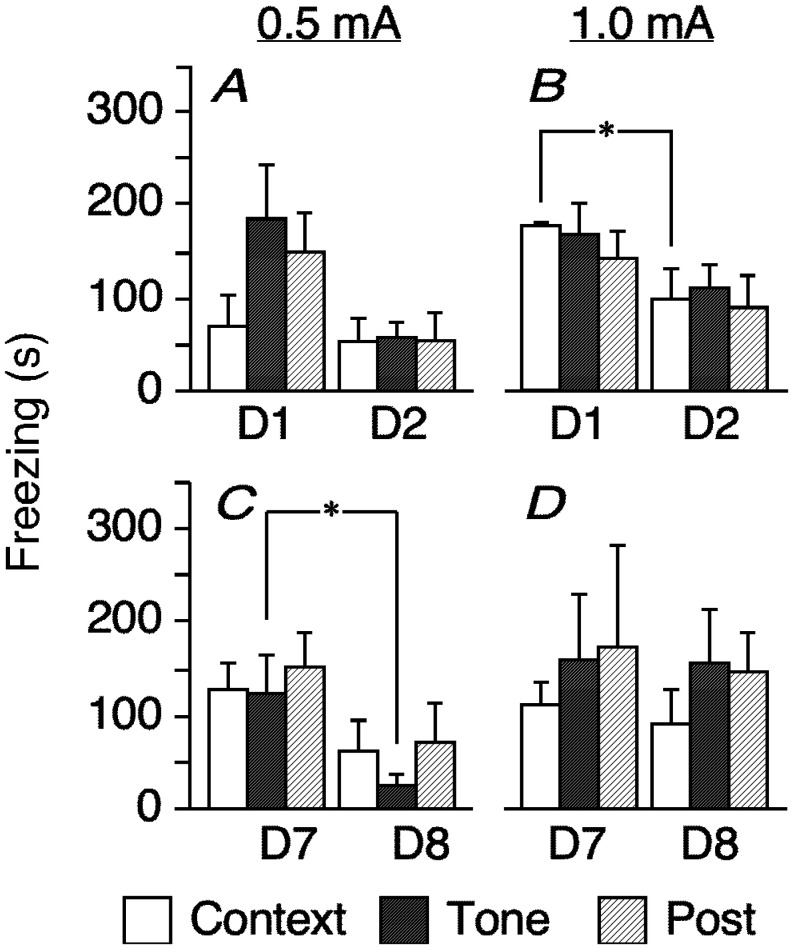
Freezing (mean ± SEM, s) in response to context or tone (3 min before or 7 min after tone start) tested on D1–D2 (conditioned at 0.5 or 1.0 mA for A and B, respectively) and on D7–D8 (conditioned at 0.5 or 1.0 mA for C and D, respectively). Post-tone (Post) indicates freezing indexing paw motion for 6 min after the tone ended (n = 5–6; * indicates P < 0.05, post-hoc analysis following four-way ANOVA with repeated measures).

Tone alone did not increase motor activity following habituation for 3 min on D0 (Figure 1A), but the 0.5 and 1.0 mA footshocks evoked an apparent effect (2192.6 ± 299.2 and 2866.8 ± 151.0 mV/kg/min, n = 12 and 11, respectively; t_21_ = −1.956, P = 0.064). Motor activity changed over time for each group (Figure 1B). Motor activity also varied significantly across the groups for sequential periods of context, tone, and the two post-tone periods (3 min for each post-tone period; F_2,29_ = 4.287, P = 0.0234, four-way ANOVA with repeated measures across two between-subject factors (shock intensity and interval day to test) and two within-subject factors (test session and test-retest day) (Figure 1C,D). A significant interaction was observed between shock intensity and test-retest day (F_2,29_ = 6.115, P = 0.0061). Motor activity also significantly varied across interval day to test (F_1,29_ = 9.586, P = 0.0043) and test session (F_3,87_ = 9.901, P = 0.0005). Relative to the control group, motor activity was significantly suppressed in both the 0.5 and 1.0 mA groups during the tone period on D1 [P = 0.0225 and 0.0303, respectively, Fisher's partial least-squares difference (PLSD)] and during the context, tone, and first post-tone periods on D7 (0.5 mA: P = 0.0148, 0.0009, and 0.0103, respectively; 1.0 mA: P = 0.0083, 0.0016, and 0.0144, respectively). Moreover, in the 1.0 mA group, sustained motor suppression occurred during the context and tone periods on D8 compared to D7 (P = 0.0348 and 0.0403). Test repetition reduced motor activity (at both footshock intensities) on D2 and (at 0.5 mA only) on D8 (Figure 1C,D).

**Figure 3.**
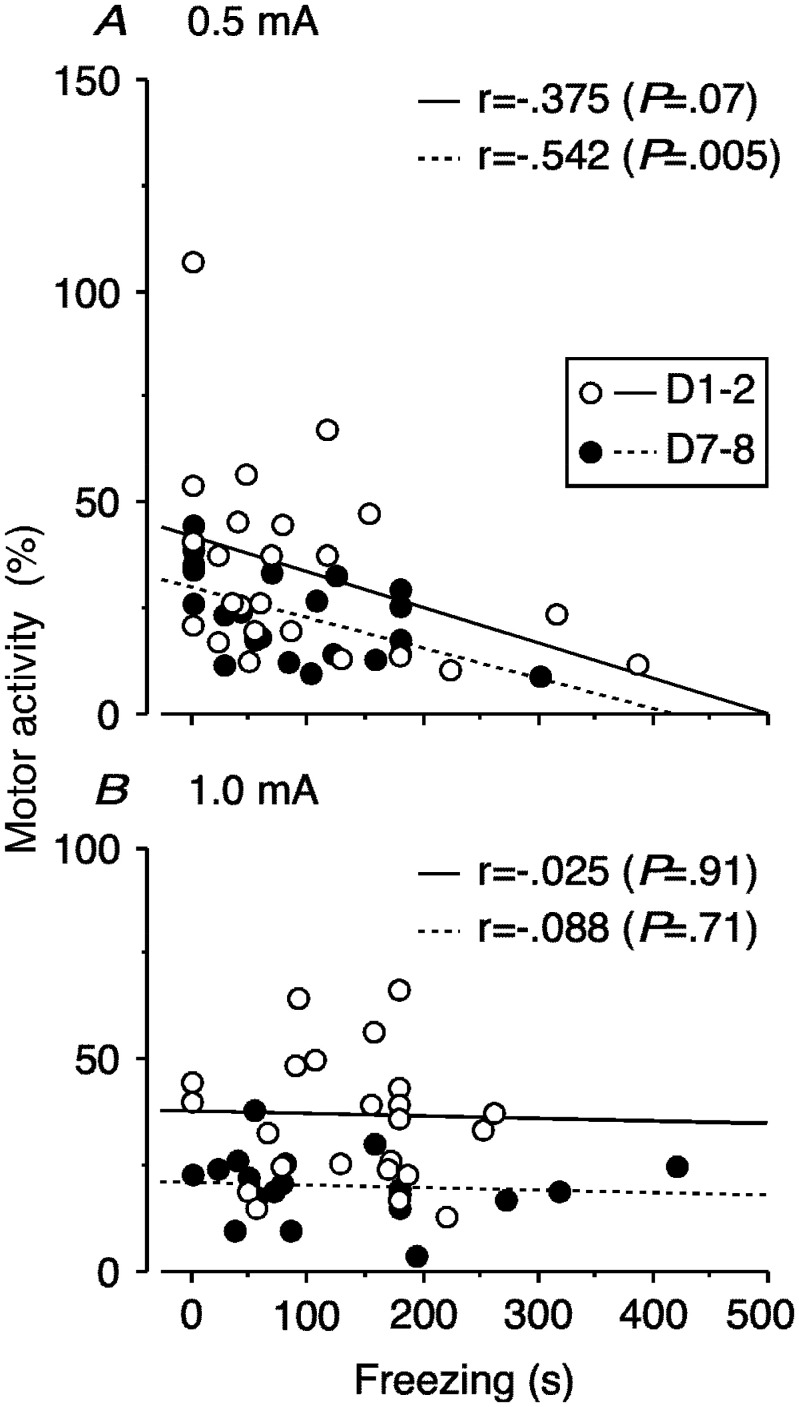
Regression analysis between responses of motor activity period and freezing epoch for context or tone on D1–D2 and D7–D8 after conditioning at 0.5 or 1.0 mA (A or B, respectively). Each circle represents an individual date pair as indicated.

Paralleling the changes in motor activity, the freezing response also varied significantly in the shock groups across freezing epochs for test session and test-retest days (F_2,44_ = 7.847 and F_1,22_ = 16.559, P = 0.0012 and 0.0005, respectively, one-way ANOVA with repeated measures). By contrast, the control group did not freeze. Contextual freezing increased with intensity on D1. In the 1.0 mA group, freezing was significantly reduced across the D1–D2 repetition (P = 0.0479, Fisher's PLSD, Figure 2A,B). Tone-cued freezing similarly increased with 0.5 and 1.0 mA footshocks on D7 and was significantly reduced in the 0.5 mA group from D7 to D8 (P = 0.0320). In the 1.0 mA group (Figure 2C,D), however, freezing increased and was sustained in each epoch from D7 to D8. Fear conditioned suppression of motor activity and freezing varied by testing interval (Figures 1, 2). With regard to context and tone, these behavioral indexes were negatively correlated at 0.5 mA but not at 1.0 mA, regardless of interval. Regression lines were lower on D7–D8 than on D1–D2 for each footshock intensity level (Figure 3).

### 3.2. Effects of lesions on motor activity and freezing across test sessions and on test-retest days

On the day following conditioning, lesioned and sham animals were separated into the following nine groups: unilateral lesion groups (the amygdala, the hippocampus, and the mPFC; n = 5/6, 6/6, and 6/6, respectively), bilateral lesion groups (amygdala, hippocampus, and mPFC; n = 6/6, 6/5, and 5/6, respectively), and disconnection groups (amygdale-hippocampus, amygdale-mPFC, and hippocampus-mPFC pairs; n = 8/8, 8/8, and 8/8, respectively).

**Figure 4.**
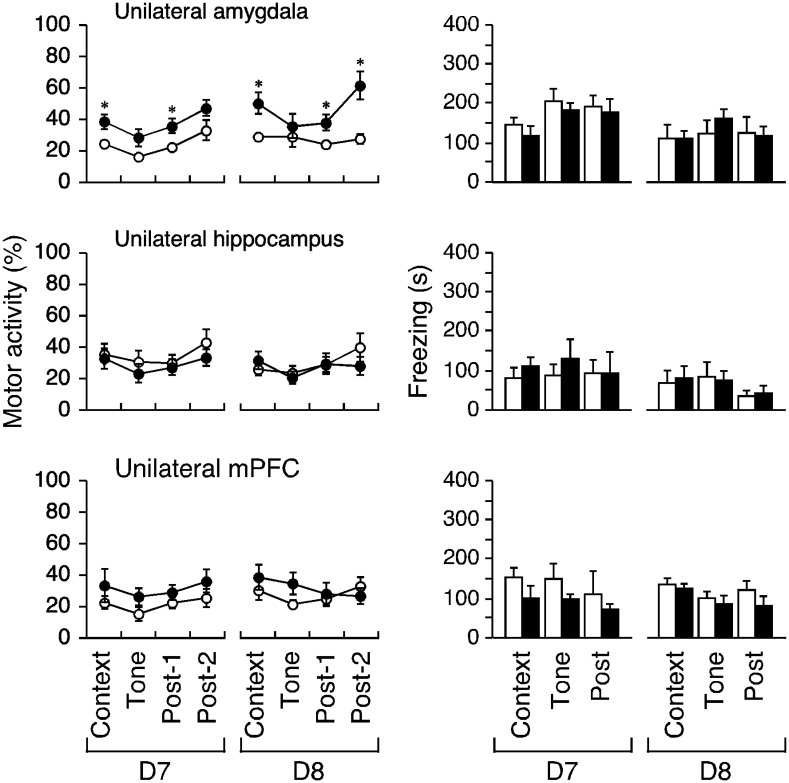
Effects of the unilateral lesions on motor activity and freezing. Motor activity (mean ± SEM, percent of motor activity during the 3 min acclimation period on the conditioning day) during context, tone, and post-tone (Post) were simultaneously measured. Open and closed circles/bars indicate sham and lesion, respectively (* indicates P < 0.05, post-hoc test following four-way ANOVA with repeated measures).

Unexpectedly, a unilateral lesion in the amygdala significantly restored motor activity suppression, as revealed by three-way repeated-measures ANOVA (lesion/sham × test/retest day × period; F_1,9_ = 12.171, P = 0.0068). The three-way interaction was statistically significant (F_3,27_ = 3.321, P = 0.0346). The post-hoc test revealed that the suppressed motor activity was significantly restored during the context and first post-tone periods on D7 (P = 0.0188 and 0.0267, respectively, Fisher's PLSD) and D8 (P = 0.0096 and 0.0138, respectively), but not during the tone period (P = 0.0524 and 0.5047, respectively, Figure 4, left-top). Unilateral hippocampal or mPFC lesions did not affect motor activity; F_1,10_ = 0.332 or 1.627 (P > 0.2 for each, Figure 4, left-middle, -bottom). The post-hoc test showed that the lesion effect on motor activity was not significant for any brain region at any time on D7–D8 (P = 0.1005–0.9761).

**Figure 5.**
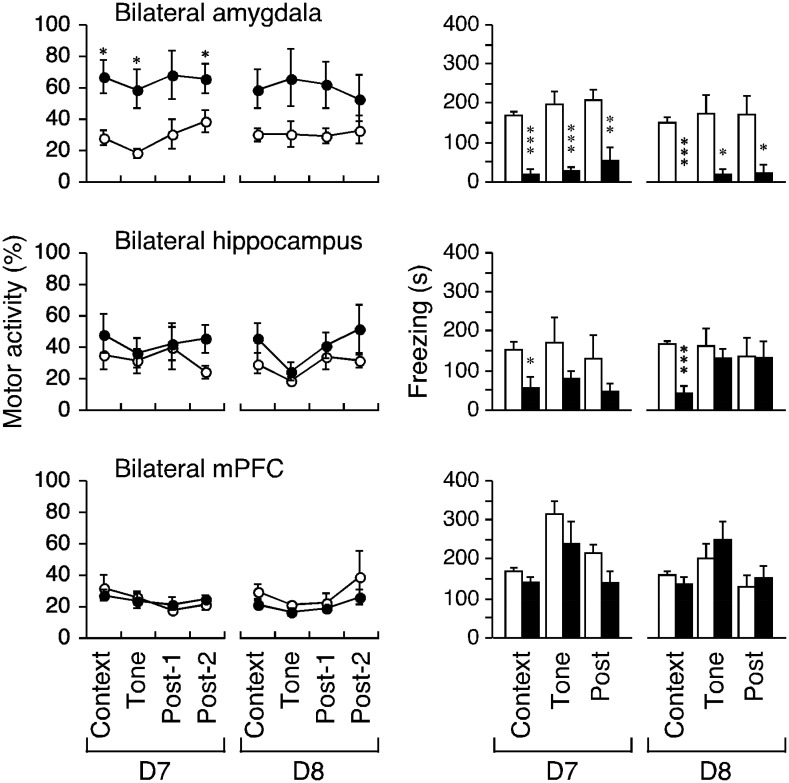
Effects of the bilateral lesions on motor activity and freezing. Motor activity during the indicated periods following bilateral lesions of the amygdale, hippocampus, or mPFC is shown. Freezing for the events was simultaneously measured. Open and closed circles/bars indicate sham and lesion, respectively (*, ** and *** indicate P < 0.05, 0.005 and 0.0005 respectively, post-hoc test following four-way ANOVA with repeated measures).

On freezing, the unilateral effect of an amygdala, hippocampal, or mPFC lesion was not statistically significant according to the three-way ANOVA (F_1,9_ = 2.976, 1.037 and 0.079, respectively, all P > 0.1). The lesions did not affect freezing on D7–D8 (P = 0.1146–0.9402, Figure 4, right).

Bilateral amygdala lesions significantly restored motor activity suppression (F_1,10_ = 7.58, P = 0.0204); the three-way interaction term was not significant (P > 0.9). The post-hoc test revealed restoration of motor activity in the context, tone, and second post-tone periods on D7 (P = 0.0060, 0.0098, and 0.0475, respectively, Figure 5, left) but not during the first post-tone period (P = 0.0594). The effect was not significant on D8 (P = 0.0537–0.2769). Bilateral lesions of the hippocampus and mPFC did not restore motor activity (both F_1,9_ > 1.1, P > 0.3, Figure 5, left-middle-bottom), but instead tended to further suppress it on D8 in rats with bilateral mPFC lesions. In the post-hoc tests, P = 0.0812–0.9169 for the hippocampus and P = 0.1669–0.7335 for the mPFC.

The bilateral amygdala lesions significantly decreased the freezing responses (F_1,10_ = 30.461, P = 0.0003); the three-way interaction term was not significant (P > 0.5). The post-hoc test revealed that the freezing response reductions for all epochs on D7 and D8 were significant (P < 0.0001–0.0178, Figure 5, right-top), but bilateral hippocampal lesions reduced only contextual freezing (P = 0.0255 and 0.0003, respectively; P = 0.1604–0.7924, Figure 5, right-middle); the three-way interaction term was significant (F_2,19_ = 5.541, P = 0.0133) but the lesion effect was not (F_1,9_ = 3.561, P = 0.0918). The bilateral mPFC lesions had no effect on freezing (F_1,9_ = 0.78, P = 0.4001), and there were no changes during any epoch on D7 or D8 (P = 0.0765–0.5932, post-hoc test).

**Figure 6.**
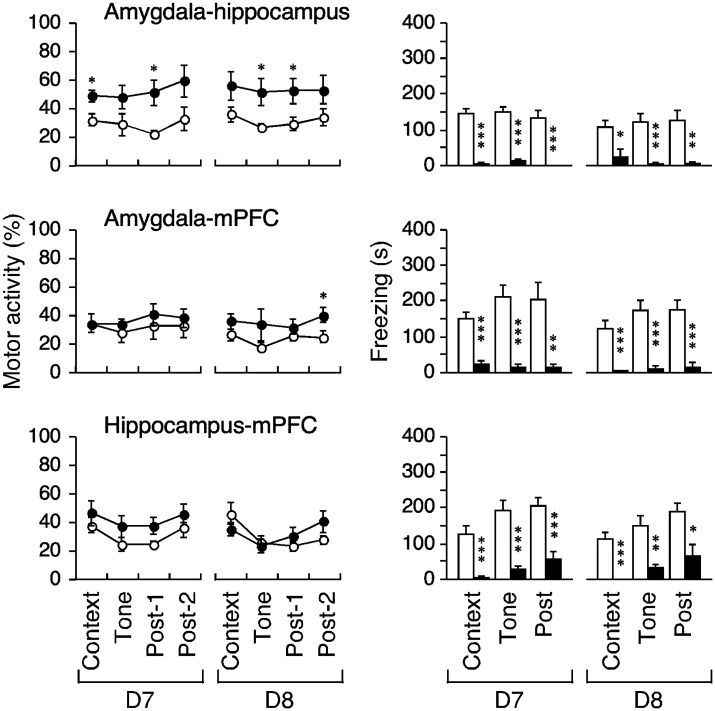
Effects of the bilateral lesions on motor activity and freezing. Motor activity during the indicated periods following crossed unilateral lesions of the amygdale-hippocampus, amygdale-mPFC, and hippocampus-mPFC is shown. Freezing for the events was simultaneously measured. Open and closed cirles/bars indicate sham and lesion, respectively (*, ** and *** indicate P < 0.05, 0.005 and 0.0005 respectively, post-hoc test following four-way ANOVA with repeated measures).

In combined amygdale-hippocampus lesioned animals, the suppressed motor activity was restored (F_1,14_ = 10.713, P = 0.0056); significant changes were evident during the context, tone, and both post-tone periods on D7 and D8 (P = 0.0098–0.0427, post-hoc test, Figure 6, left). Following the creation of amygdale-mPFC lesions, the suppressed motor activity was restored only during the second post-tone period on D8 (P = 0.0377, post-hoc test); three-way repeated-measures ANOVA did not detect a significant effect (F_1,14_ = 1.526, P = 0.2371). The hippocampal-mPFC lesion group did not show suppressed motor activity (F_1,14_ = 1.205, P = 0.2908) during any periods on D7–D8 (P = 0.0551–0.6630, post-hoc test).

In all disconnection groups, freezing was significantly reduced (all F_1,14_ > 30, P < 0.0001) during all epochs on D7–D8 (P ≤ 0.0103, Figure 6, right). Notably, only the amygdale-mPFC lesion group showed stereotypy, as demonstrated by horizontal head weaving at approximately 0.5 Hz during tests. Stereotypy may have reduced the propensity to freeze and may have affected certain other cognitive attributes. The other groups showed no atypical behavior.

The motor activities of the sham control group across all brain regions (mean ± SEM) during the context, tone, and two post-tone (Post-1 and Post-2) periods were 31.5 ± 1.7, 24.4 ± 2.1, 27.2 ± 2.3, and 32.0 ± 2.4 mV/kg/min on D7; and 31.5 ± 2.1, 24.0 ± 1.5, 27.2 ± 1.2, and 32.4 ± 1.7 mV/kg/min on D8, respectively. The freezing values (mean ± SEM) during the context, tone, and post-tone periods were 144.3 ± 8.8, 187.2 ± 20.5, and 174.2 ± 21.1 s on D7; and 127.9 ± 8.4, 155.9 ± 11.1, and 145.9 ± 13.9 s on D8.

### 3.3. Histology

**Figure 7.**
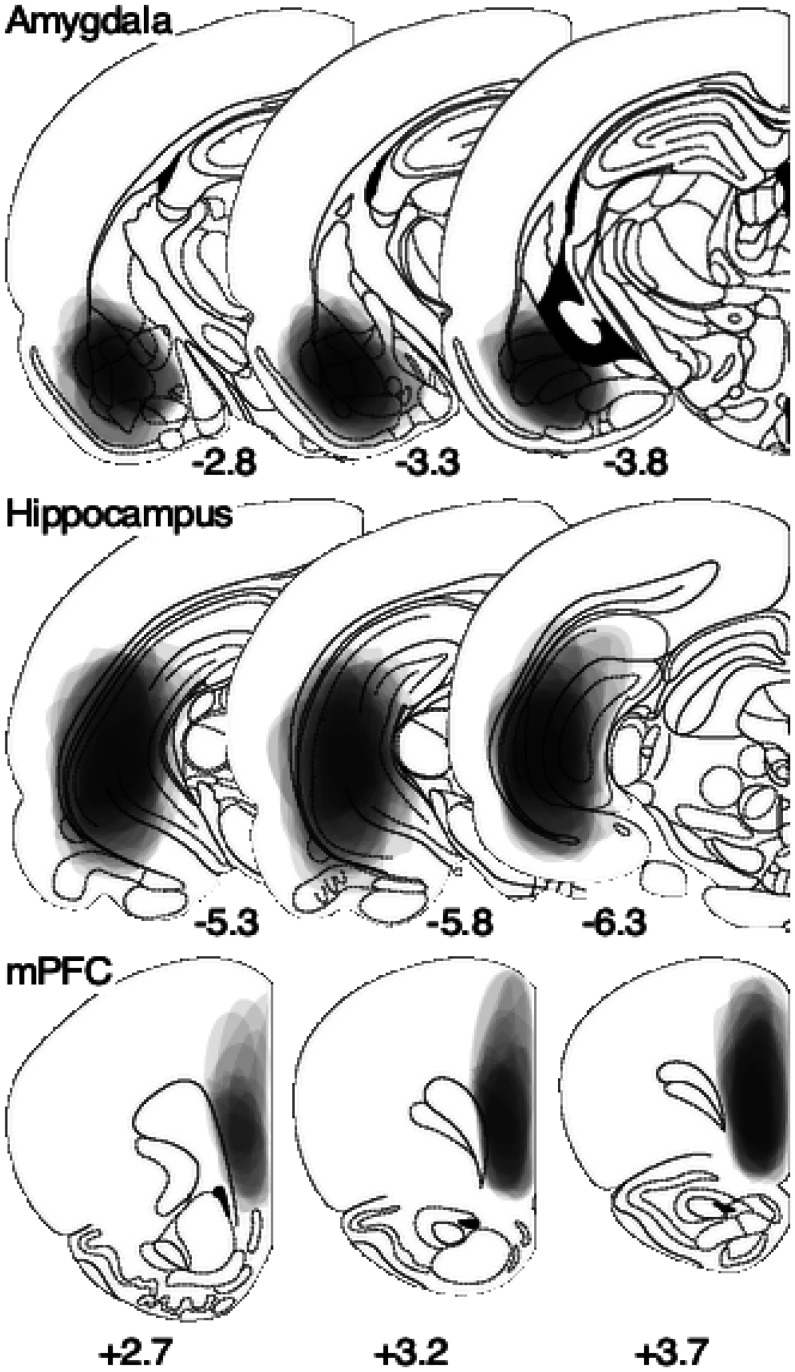
Ipsilaterally superimposed representations of lesion locations in the basolateral/basomedial amygdale, caudal/ventral hippocampus, and mPFC. Transparent 5% gray images of each lesion are superimposed on the brain sections indicate the relative distance (in mm) from the bregma.

The distribution of lesioned areas in the amygdala, hippocampus, and mPFC was confirmed histologically. The amygdala lesions covered mainly the lateral (ventro), basolateral, and basomedial areas, the hippocampal lesions covered the caudal/ventral area, and the mPFC lesions covered the prelimbic/infralimbic area (Figure 7).

## 4. Discussion and conclusions

Here, we tested a procedure showing persistent responses [hypoactivity (i.e., motor activity reduction) and freezing] related to fear conditioning. Our lesion studies indicated that neurocircuit controls between hypoactivity and freezing differed among the amygdala, hippocampus, and mPFC, implying that several different types of medicine may be necessary for patients with PTSD [10].

### 4.1. Characteristics of memory persistence after fear conditioning

Fear conditioning differentially influenced motor activity and freezing across footshock intensity and test-retest interval (Figures 1 and 2). We performed serial context (background cue) and tone (explicit cue) tests. While suppression of motor activity to context did not vary across footshock intensity relative to the control on D1–D2 (Figure 1C, left), contextual freezing was longer at the 1.0 mA footshock intensity level than at the 0.5 mA level (Figure 2A,B). The subsequent tone-cued freezing was relatively long at either intensity. Freezing was more evident for tone than context in lower intensity conditioning, as described previously [6]; c.f., “generalization”, [42]. Therefore, motor activity and freezing were negatively correlated at 0.5 mA but not at 1.0 mA (Figure 3).

For the longer test interval (D7–D8), motor activity was significantly suppressed, regardless of footshock intensity, from context to the first post-tone period at D7. From context to tone on test repetition on D8, motor activity was suppressed at 1.0 mA (Figure 1D). After conditioning at 1.0 mA, freezing increased for either epoch from D7–D8 and changed from increasing to decreasing freezing for context from D1 to D2. However, conditioning at 0.5 mA resulted in decreased freezing for tone from D7 to D8 (Figure 2). Such effects of interval length are consistent with memory-based consolidation because learning takes several days or weeks in most memory systems [43],[44]. In other words, an “incubation” period is required [45],[46]. The relationship between the interval length and the shock intensity affected the relationship between motor activity and freezing (Figure 3); the *y* intercept of the regression line was smaller on D7–D8 than on D1–D2 for both footshock intensities. These results suggest that the consolidation reduced behavioral flexibility and resources, as reported in a previous study of PTSD [47].

Although conditioned suppression of motor activity and freezing was reportedly correlated after fear conditioning [48], freezing could be dissociated from the conditioned suppression of motor activity following generation of lesions of the periaqueductal gray, which was a downstream process of the amygdala [49]. Our results also imply that such a correlation could be allowed under limited conditions; a synergistic effect between the intensity of the unconditioned stimulus and the interval to test (1.0 mA on D7–D8) did not allow such a correlation. These results suggest that at least two types of relationship exist between ‘freezing’ and ‘other-than-freezing’ motor activity in the context of memory consolidation/persistence.

### 4.2. Effects of lesions on sequential memory control after fear conditioning

Nine lesion conditions, introduced in the amygdala, hippocampus, and mPFC, disconnected and maintained pathways of nine connections (three bilateral connections and three connections across each hemisphere). These lesion conditions exhibited a number of different effects on motor activity and freezing (Figures 4–6). An equivalent effect of restoration on both hypoactivity and freezing was evident in the bilateral amygdala lesion (Figure 5, top), as in previous reports on conditioned fear (e.g., [6]). However, neither the two unilateral lesion groups (with lesions in the hippocampus and the mPFC) nor the bilateral mPFC lesion group exhibited motor activity reduction or freezing (Figure 4, middle and bottom, Figure 5, bottom).

The unilateral amygdala lesion effectively restored the reduced motor activity, but not freezing (Figure 4, top). The restored levels of motor activity seemed to be similar between rats with versus without contralateral hippocampal lesions (Figure 6, right-top or Figure 4, right-top). Indeed, when the restorative effect of the bilateral amygdala lesions on hypoactivity was taken to be 100% in total (4 periods), the effects of a unilateral lesion of the amygdala and the amygdala-hippocampus lesion scored 58% and 68% respectively. In addition, the bilateral hippocampal lesions had no significant effect on hypoactivity (Figure 5, left-middle). These data implied that the hippocampus did not directly control hypoactivity. Also, the restorative effect of a unilateral amygdala lesion on hypoactivity almost disappeared when a contralateral mPFC lesion was created (Figure 4, right-top vs. Figure 6, right-top), whereas unilateral mPFC lesions alone had no significant effect (Figure 4, left-bottom). These results cannot be clearly attributed to the addition or subtraction of lesional effects and we speculate that the amygdala may play dual roles in memory consolidation (following the fear-conditioning paradigm) of freezing and suppression of other-than-freezing activity.

Regarding the bilateral amygdala lesions: During the context, tone, and post-2 D7 period, the reduced motor activity was significantly restored, with similar trends evident on D8; freezing was rescued during all episodes on both days (Figure 5, top). Restoration of motor activity reduction was weaker on D8, possibly reflecting the involvement of areas other than the amygdala, such as the hippocampus and mPFC, as described above. Such weakened restoration was not apparent in rats with unilateral amygdala lesions (Figure 4, left-top), which were inefficient during tone delivery on either day. In other words, the remaining (intact) unilateral amygdala likely received inhibitory signals (triggering the reduction) from the mPFC rather than the hippocampus. The inhibitory control from the mPFC to the amygdala may correspond to habitual behaviors under the implicit condition (e.g., context) rather than occasional behaviors under the explicit condition (e.g., conditioned stimulus).

Conversely, the amygdala, by way of the periaqueductal gray [51],[52], must have been directly involved in freezing triggered by emotional memory (Figure 5, top right). Across all episodes, three disconnection versions reproduced the bilateral amygdala lesion effects on freezing (Figure 6, right). The bilateral hippocampus lesions rescued freezing in response to context, but not tone, on test-retest, similar to previous studies [6]. The connection between the amygdala and the hippocampus appear to be essential for this rescue, as described previously [53]. The mPFC may be rendered hypofunctional by stress, which involves the hippocampal-prefrontal pathway [53]; this may explain freezing restoration by the disconnection of the hippocampus-mPFC (Figure 6, right-bottom). Rats with bilateral lesions of the hippocampus retained the mPFC-amygdala pathways, and those with bilateral lesions of the mPFC retained the hippocampal-amygdala pathways in both hemispheres; therefore, these pathways may be involved in persistent freezing (Figure 5, right-middle and -bottom). Three disconnection versions, one unilateral and another contralateral lesions out of three regions, would disrupt these pathways (Figure 6, right).
